# The Impact of Emotional Arousal on Physical Pain and Numbness in Individuals Who Engage in Non‐Suicidal Self‐Injury

**DOI:** 10.1111/sltb.70128

**Published:** 2026-07-09

**Authors:** Michelle K. Hiner, Teresa M. Leyro, Evan M. Kleiman, Amy Kranzler, Edward A. Selby

**Affiliations:** ^1^ Department of Psychology Rutgers University New Brunswick New Jersey USA; ^2^ Handspring Health Jersey City New Jersey USA

**Keywords:** ecological momentary assessment, emotional arousal, non‐suicidal self‐injury, numbness, pain

## Abstract

**Introduction:**

Non‐suicidal self‐injury (NSSI) is commonly understood as a behavior that serves to regulate affect, yet less is known about how specific emotional states relate to physical sensations during NSSI. This study examined whether high‐arousal negative emotions (HANEs) and low‐arousal negative emotions (LANEs) before NSSI were differentially associated with physical pain and numbness during NSSI episodes.

**Methods:**

Forty adolescents and emerging adults with recent NSSI completed a 14‐day ecological momentary assessment protocol. Across 143 NSSI episodes, participants reported negative emotions immediately before NSSI and physical pain and numbness during NSSI. Linear mixed‐effects models separated within‐person emotion fluctuations from between‐person differences.

**Results:**

At the between‐person level, individuals who reported higher average HANEs before NSSI reported greater pain and lower numbness during NSSI, whereas individuals who reported higher average LANEs reported lower pain and greater numbness. At the within‐person level, episodes preceded by higher‐than‐usual LANEs were associated with greater pain, but no within‐person emotion predictors were significantly associated with numbness.

**Conclusion:**

Findings suggest that high‐ and low‐arousal negative emotions may correspond to distinct somatic experiences during NSSI, highlighting the importance of assessing both emotional antecedents and physical sensations during self‐injury.

Non‐suicidal self‐injury (NSSI), defined as the direct and intentional destruction of one's own bodily tissue without suicidal intent, is a robust predictor for suicide attempts and a significant public health concern (Franklin et al. 2017; International Society for the Study of Self‐Injury 2022; Nock and Favazza 2009). Individuals who engage in NSSI report their experiences of negative emotions as more frequent and more intense than those without a history of self‐injury (Anderson and Crowther 2012; Victor and Klonsky 2014). The most commonly endorsed function of NSSI is affect regulation, or using self‐injury to alleviate negative emotional states (Taylor et al. 2018). For some, NSSI may serve to replace, redirect, or regulate emotional distress through physical sensation or pain. Consistent with the clinical importance of pain in NSSI, associations have been found between diminished physical pain during self‐injury and suicidal behavior (Ammerman et al. 2016; Franklin et al. 2012; Hepp et al. 2026). Despite this, relatively little is known about how specific emotional states relate to the physical sensations experienced during NSSI itself, including both physical pain and physical numbness.

Physical pain is typically defined as an unpleasant sensory and emotional experience associated with actual or potential tissue damage (Raja et al. 2020). From an evolutionary perspective, acute pain serves an adaptive function by alerting individuals to bodily threat and motivating behaviors that prevent further injury (Lumley et al. 2011; Walters and Williams 2019). In the context of NSSI, however, this protective function becomes more complex, as bodily injury may be intentionally used to regulate emotional distress. Laboratory studies suggest that individuals with a history of NSSI often demonstrate altered pain processing compared to individuals without NSSI histories, including higher pain thresholds, greater pain tolerance, and lower subjective pain intensity in response to painful stimuli (Koenig et al. 2016). These findings suggest that physical pain during NSSI may not operate solely as an aversive signal of bodily threat, but may instead be embedded within broader affective, regulatory, and somatosensory processes.

In addition to pain, individuals who self‐injure frequently report experiences of physical numbness, defined as hyposensitivity or absence of somatic sensation, though sometimes associated with tingling or “pins‐and‐needles” sensations (Cummins et al. 2021; Freedman 2023; Horne and Csipke 2009; Westfall and Veilleux 2026). Physical pain and numbness also should not be assumed to represent opposite ends of a single continuum. Pain and numbness can co‐occur in other somatosensory conditions, such as neuropathic pain (Dworkin et al. 2003; Flowers et al. 2021). Evidence suggests that pain sensitivity is reduced among individuals who self‐injure, but physical numbness during NSSI remains comparatively understudied (Bohus et al. 2000; Kirtley et al. 2016; Koenig et al. 2016). Thus, examining both pain and numbness may provide a more nuanced understanding of the bodily experience of NSSI. In the present study, numbness refers specifically to physical or bodily numbness during NSSI, rather than emotional numbness, blunting, or emptiness.

A substantial body of research supports the role of negative affect in the onset and maintenance of NSSI. Ecological momentary assessment (EMA) studies, which assess experiences in real time or near‐real time in naturalistic contexts, have shown that increases in negative affect prospectively predict self‐injurious thoughts and behaviors (Brown et al. 2022; Kuehn et al. 2022). However, negative affect is often treated as a broad, unitary construct (e.g., Rodríguez‐Blanco et al. 2018). This approach may obscure meaningful differences among negative emotional states. For example, anxiety and loneliness both involve negative valence, but these states differ in arousal and bodily experience (Nummenmaa et al. 2014). These distinctions may be especially important for understanding pain and numbness during NSSI.

The relationship between affect and pain is complex and not uniformly directional. Theoretical models suggest that negative affect can both amplify and inhibit pain through distinct physiological and cognitive mechanisms. For example, heightened autonomic arousal, muscle tension, and hypervigilance may increase pain sensitivity, whereas endogenous opioid release and attentional disengagement may reduce pain perception (see Janssen 2002). These mechanisms are considered adaptive in acute contexts but may contribute to sensitization and functional impairment when persistently engaged (Rhudy et al. 2010).

Consistent with this perspective, experimental research has demonstrated that emotional states systematically modulate pain perception and nociceptive responding. For instance, paradigms such as the Emotional Controls of Nociception (ECON) have shown that unpleasant emotional states enhance both subjective pain and spinal nociceptive reflexes, whereas pleasant states inhibit them (Rhudy et al. 2010). Notably, these effects appear to persist across repeated stimulus presentations, suggesting that emotional modulation of pain may operate independently of habituation to repeated nociceptive stimuli, a process often discussed in models of NSSI and suicide risk (Joiner 2005; Van Orden et al. 2010). Importantly, this work has largely focused on broad distinctions in emotional valence, with comparatively less attention to variability within negatively‐valenced affect itself.

Although negative affect is central to NSSI, treating it as a single composite may obscure meaningful differences among emotional states (Rodríguez‐Blanco et al. 2018). For instance, anger, anxiety, sadness, and loneliness are all negatively‐valenced, but differ in arousal and may therefore relate differently to bodily sensations (Russell 1980; Yik et al. 2011). This distinction may be particularly important for understanding pain and numbness during NSSI. Negative emotions of higher arousal levels are often associated with autonomic activation and heightened bodily sensation, including increased attention to internal bodily cues (Esteve and Camacho 2008; Kreibig 2010; Nummenmaa et al. 2014). In contrast, lower arousal negative emotional states may be more closely linked to withdrawal, disengagement, and diminished bodily awareness, particularly when they occur in the context of dissociation (Dewe et al. 2016; Price and Thompson 2007). This suggests that high‐ and low‐arousal negative emotions may have distinct relevance for somatosensory experiences during NSSI. However, examining each discrete emotion separately also has limitations (e.g., Barrett 2006). Negative emotions often co‐occur and individuals who engage in NSSI may have difficulty differentiating among specific emotional states (Bresin 2014; Cerutti et al. 2018; Zaki et al. 2013). Therefore, the present study used an arousal‐based approach, grouping negative emotions into high‐arousal negative emotions (HANEs; overwhelmed, anxious/afraid, angry, frustrated) and low‐arousal negative emotions (LANEs; sad, lonely, empty, hurt/rejected). Prior retrospective work has distinguished between high‐ and low‐arousal negative emotions surrounding NSSI, finding that both tend to decrease after self‐injury, with larger reductions in HANEs (Claes et al. 2010; Klonsky 2009). However, less is known about whether these arousal‐based emotional states are differentially associated with physical sensations during NSSI itself. This approach provides a theoretically grounded middle ground between broad negative affect composites and individual emotion‐level analyses, allowing us to examine whether arousal‐based differences in negative affect have distinct associations with pain and numbness during NSSI.

The current study uses EMA to examine how momentary emotional experiences directly preceding NSSI relate to reports of physical pain and numbness during self‐injury episodes in adolescents and emerging adults. By focusing on variability within negative affect, this work aims to clarify how differences in emotional arousal may differentially relate to somatosensory experiences during self‐injury.

We first examined descriptive associations among study variables. We expected high‐arousal negative emotions (HANEs) and low‐arousal negative emotions (LANEs) before NSSI episodes to be positively associated, reflecting the tendency for negative emotional states to co‐occur. We also expected physical pain and physical numbness during NSSI to be negatively associated, although this hypothesis was considered exploratory given that pain and numbness may represent distinct, rather than strictly opposing, somatosensory experiences.

Our primary hypotheses focused on whether arousal‐based negative emotion composites before NSSI were differentially associated with physical pain and physical numbness during NSSI. At the within‐person level, we hypothesized that episodes preceded by higher‐than‐usual HANEs would be associated with greater physical pain and lower physical numbness during the same NSSI episode. In contrast, we hypothesized that episodes preceded by higher‐than‐usual LANEs would be associated with lower physical pain and greater physical numbness during the same NSSI episode.

At the between‐person level, we hypothesized that individuals who generally reported higher HANEs before NSSI episodes would report greater average physical pain and lower average physical numbness during NSSI. Conversely, we hypothesized that individuals who generally reported higher LANEs before NSSI episodes would report lower average physical pain and greater average physical numbness during NSSI.

## 1 Materials and Methods

### 1.1 Participants

Participants in the broader study for which this data were collected included 47 community‐based adolescents and emerging adults who reported engaging in NSSI behaviors at least twice in the 2 weeks prior to study baseline (see Kranzler et al. 2018; Selby et al. 2018). The present study reflects secondary analyses of data from this previously reported EMA study of NSSI among adolescents and emerging adults. Study procedures were approved by the Rutgers University Institutional Review Board. All participants 18 years of age or older provided written informed consent to participate in the study, and both participant assent and parental/guardian consent were obtained from participants under 18 years of age.

The sample size was determined by the original study design rather than an a priori power analysis for the present secondary analyses. Of the total 47 participants, 40 endorsed at least one episode of NSSI during the 2 weeks of study participation (see Table 1 for demographic information of this subsample).

**TABLE 1:** Sample demographic information (*N* = 40).

Variable	*M*	SD	*n*	%
Age (in years)				
15–17	19.29	1.65	5	12.5
18–21			35	87.5
Gender				
Female			28	70.0
Male			11	27.5
Transgender			1	2.5
Sexual orientation				
Heterosexual			26	65.0
Bisexual			7	17.5
Homosexual			2	5.0
Other/unlisted			3	7.5
Unsure/“Do not wish to report”			2	5.0
Race/ethnicity				
White			15	37.5
Black			6	15.0
Asian			9	22.5
Hispanic/Latino			6	15.0
Two or more races			4	10.0

*Note:* Includes only participants in subsample who reported at least one NSSI event during the EMA period. Gender categories reflect the response options used in the original study and are not ideal by current reporting standards, as they do not fully distinguish gender identity from sex assigned at birth.

### 1.2 Procedures

#### 1.2.1 Recruitment

Participants were initially recruited for the study via flyers, online advertisements, and referrals from community‐based treatment centers, and eligible participants underwent a phone screen and baseline visit. To be included in the study, participants were required to be between 15 and 21 years of age and had to have engaged in NSSI at least twice in the 2 weeks prior to study baseline. They were excluded from the study if either of the two inclusion criteria was not met, or if they did not speak English, were deemed to be above moderate risk for suicide, or had previously been diagnosed with a psychotic disorder including any schizophrenia spectrum disorder, life‐threatening anorexia nervosa, or intellectual or developmental disability.

#### 1.2.2 Ecological Momentary Assessment

This study collected data via the *Track It!* smartphone app for Android devices. If participants did not have an Android phone, they were lent a study Android device with the *Track It!* app installed. The *Track It!* app utilized both signal‐contingent and event‐contingent assessments. Signal‐contingent assessments were prompted randomly within five predetermined time intervals throughout the day, and participants had 1 h to complete assessments after being prompted. Participants were asked to complete an event‐contingent assessment after engaging in NSSI (i.e., event‐contingent assessment). Assessments took approximately 5 min to complete. Participants were asked to participate in the study for 14 days following two “practice days,” and they were given $150 for completion of the 2‐week monitoring period, as well as the opportunity to gain another $150 for completing 80% of the signal‐contingent assessments or more.

### 1.3 Baseline Measures

#### 1.3.1 Inventory of Statements About Self‐Injury (ISAS)

The Inventory of Statements about Self‐Injury (ISAS; Klonsky and Glenn 2009) is a self‐report measure that assesses both the frequency and functions of NSSI. The first section assesses lifetime frequency of 12 NSSI methods, with total NSSI frequency calculated by summing the reported frequency across methods. The second section assesses 13 functions, or reasons, for engaging in NSSI. Participants rated the relevance of 39 statements reflecting different NSSI functions with responses ranging from 0 (*not at all relevant for me*) to 2 (*very relevant for me*). Scores for each function range from 0 to 6, with higher scores indicating higher function endorsement. For the present study, we focused specifically on the affect regulation and anti‐dissociation function subscales.

#### 1.3.2 Positive and Negative Affect Schedule (PANAS)

The Positive and Negative Affect (PANAS; Watson et al. 1988) is a 20‐item self‐report measure designed to assess the extent to which individuals experience positive and negative emotions. Although the PANAS includes two 10‐item subscales, the present study focused primarily on the Negative Affect subscale. Participants rated 10 negative affect items on a five‐point scale, with higher summed scores indicating greater negative affect.

#### 1.3.3 Mini International Neuropsychiatric Interview (MINI)

The Mini International Neuropsychiatric Interview (MINI; Sheehan et al. 1998) is a brief, structured diagnostic interview designed to assess DSM‐IV‐TR psychiatric symptoms and related clinical risk factors. For the present study, we focused specifically on item C9, which assesses participants' history of suicide attempts. 10 individuals (25.0%) in the subsample of participants who reported NSSI during the EMA period reported at least one lifetime suicide attempt.

### 1.4 Ecological Momentary Assessment Measures

#### 1.4.1 Assessment of Physical Pain and Numbness Intensity During Active NSSI

If NSSI behavior was endorsed at any assessment period, participants were asked to report their experience of physical pain before, during, and after the NSSI episode. Pain was rated on a scale from 0 (no pain) to 10 (extremely painful). Similarly, physical numbness was assessed before, during, and after the NSSI episode using the same scale from 0 (no numbness) to 10 (extreme numbness). In the present study, only pain and numbness reported during the active phase of NSSI were analyzed, while ratings of these variables before and after each NSSI episode were not examined.

#### 1.4.2 Assessment of Negative Emotion Intensity Prior to NSSI

Twenty‐one emotions were evaluated in each assessment, 11 of which were considered negative emotions. Emotions were asked about in the present moment at all momentary assessment points. Additionally, if an NSSI behavior was endorsed during any assessment period, items relating to emotion intensity before, during, and after the NSSI were displayed. Participants were asked how intensely they felt each emotion at the given timepoint, and answers were measured on a scale from 0 (not at all) to 10 (extremely).

#### 1.4.3 Construction of High‐ and Low‐Arousal Negative Emotion Composites

HANE scores were calculated by averaging momentary intensity ratings of overwhelmed, anxious/afraid, angry, and frustrated immediately before NSSI. LANE scores were calculated by averaging momentary ratings of sad, lonely, empty, and hurt/rejected immediately before NSSI. Higher scores reflected greater intensity of each arousal‐based negative emotion composite prior to NSSI.

### 1.5 Data Analytic Plan

Data analyses were conducted in *R* (version 4.4.3) using the *psych, tidyverse*, *lme4*, *lmerTest*, and *broom.mixed* packages. Analyses were limited to EMA observations in which participants reported NSSI and had complete data on the primary predictors and outcomes. For each NSSI episode, HANE scores were calculated by averaging ratings of overwhelmed, anxious/afraid, angry, and frustrated immediately before self‐injury. LANE scores were calculated by averaging ratings of lonely, sad, empty, and hurt/rejected immediately before self‐injury. Physical pain and numbness during NSSI were analyzed using participants’ retrospective ratings of these sensations during the self‐injury episode.

An exploratory factor analysis (EFA) was conducted using the 11 negative emotion items assessed across EMA prompts to inform construction of the HANE and LANE composites. Data suitability was evaluated using the Kaiser‐Meyer‐Olkin measure and Bartlett’s test of sphericity. Parallel analysis informed factor retention, and a maximum likelihood EFA with oblimin rotation was estimated. The EFA was used descriptively to examine whether negative emotion items grouped in a manner broadly consistent with the proposed arousal‐based distinction; primary analyses used observed composite scores rather than latent factors.

Person‐level descriptive statistics were calculated for average physical pain, physical numbness, HANE, and LANE scores across NSSI episodes, as well as affect regulation function, anti‐dissociation function, lifetime NSSI frequency, and baseline negative affect. Spearman correlations were used to examine bivariate associations among person‐level variables and were estimated using pairwise complete observations.

Primary hypotheses were tested using two linear mixed‐effects models: one predicting physical pain during NSSI and one predicting physical numbness during NSSI. HANE and LANE scores were decomposed into within‐person and between‐person components. Within‐person predictors reflected episode‐level deviations from each participant’s own average HANE or LANE score, whereas between‐person predictors reflected each participant’s average HANE or LANE score across NSSI episodes. Both models included fixed effects for within‐ and between‐person HANE and LANE scores and a random intercept for participant to account for nesting of NSSI episodes within individuals. Models were estimated using restricted maximum likelihood, and statistical significance and 95% confidence intervals were calculated.

## 2 Results

Across the sample, participants submitted 3357 EMA observations, 86.8% of which were signal‐contingent prompted surveys (*n* = 2916) and 13.1% of which were self‐initiated/event‐contingent surveys (*n* = 441). Participants completed an average of 71.4 surveys across the EMA period (SD = 16.6). No participants withdrew from the study, and 83.0% demonstrated good compliance defined as completing at least 80% of prompted signal‐contingent assessments. Of all completed surveys, 145 included report of NSSI behavior. Analyses used complete cases for NSSI episodes with available pre‐NSSI emotion and during‐NSSI pain/numbness ratings, which excluded two observations thus leaving an analytic sample of 143 NSSI observations from 40 participants. Participants in this subsample reported an average of 3.63 NSSI episodes during the two‐week EMA period (SD = 2.83; IQR = [2, 5], range = 1–15).

Because the present study used observed arousal‐based emotion composites rather than latent variables, an EFA was used descriptively to evaluate whether the negative emotion items grouped in a manner broadly consistent with the proposed HANE/LANE distinction. The correlation matrix was appropriate for factor analysis, with an overall Kaiser‐Meyer‐Olkin measure of sampling adequacy of 0.88, item‐level MSAs ranging from 0.84 to 0.90, and a significant Bartlett's test of sphericity, *χ* ^2^(55) = 14505.62, *p* < 0.001. A maximum likelihood EFA with oblimin rotation supported a four‐factor solution. Although the chi‐square test of model fit was significant, *χ* ^2^(17) = 172.70, *p* < 0.001, other fit indices suggested acceptable to good fit, including RMSR = 0.01, TLI = 0.965, and RMSEA = 0.052, 90% CI [0.045, 0.059]. Factor correlations ranged from 0.38 to 0.54, indicating that the factors were related but distinguishable. The EFA supported a coherent LANE factor, whereas high‐arousal emotions separated into anxiety/overwhelm and anger/frustration factors (see Table 2). Because both sets of items reflected high‐arousal negative affect and were central to the study's arousal‐based hypotheses, they were combined into a single HANE composite. A separate factor comprised of ashamed, guilty, and embarrassed reflecting self‐conscious emotions were excluded from the HANE/LANE composite (see Factor 1, Table 2).

**TABLE 2:** Factor loadings from exploratory factor analysis of negative emotion items.

Item	Factor loading				Communality
	1	2	3	4	
Ashamed	**0.814**	0.054	0.054	−0.050	0.719
Guilty	**0.762**	−0.029	0.024	−0.007	0.574
Embarrassed	**0.625**	−0.017	−0.041	0.228	0.542
Lonely	−0.054	**0.787**	−0.032	0.022	0.574
Sad	0.072	**0.621**	0.263	−0.042	0.641
Empty	0.060	**0.556**	−0.032	−0.068	0.299
Hurt/rejected	0.141	**0.491**	−0.123	0.277	0.467
Overwhelmed	0.016	0.040	**0.757**	0.069	0.665
Anxious/afraid	0.167	0.029	**0.645**	−0.035	0.560
Angry	0.137	0.061	0.036	**0.676**	0.641
Frustrated	−0.036	0.052	**0.411**	**0.488**	0.567

*Note:* The exploratory factor analysis was used descriptively to inform construction of observed emotion composites rather than to test a confirmatory latent measurement model. High‐arousal negative emotion (HANE) items included overwhelmed, anxious/afraid, angry, and frustrated. Low‐arousal negative emotion (LANE) items included sad, lonely, empty, and hurt/rejected. Shame, guilt, and embarrassment were excluded from primary composites because they loaded on a distinct self‐conscious emotion factor. Factor loadings ≥ 0.40 are bolded. Extraction method = maximum likelihood; rotation = oblimin. Communalities (*h* ^2^) are presented in the final column.

### 2.1 Descriptive Statistics and Correlations

Person‐level descriptive statistics and bivariate correlations are presented in Table 3. Average physical pain and physical numbness during NSSI were moderate in intensity and were not significantly correlated with one another. Physical pain during NSSI was positively associated with average pre‐NSSI HANE intensity, whereas physical numbness during NSSI was positively associated with average pre‐NSSI LANE intensity. Physical pain was negatively associated with anti‐dissociation function endorsement and baseline negative affect, while physical numbness was positively associated with both anti‐dissociation function endorsement and baseline negative affect. HANE and LANE intensity were positively associated with one another, indicating that individuals with more intense average HANEs before NSSI also tended to report more intense LANEs. Notably, LANE intensity and anti‐dissociation function endorsement were each positively associated with history of suicide attempt, and anti‐dissociation function endorsement was also positively associated with lifetime NSSI frequency. Affect regulation function endorsement was not significantly associated with any person‐level study variables.

**TABLE 3:** Person‐level descriptive statistics and spearman correlations among study variables.

	*M*	SD	1	2	3	4	5	6	7	8	9
1. Physical pain^a^	4.14	1.70	—								
2. Physical numbness^a^	3.86	2.65	−0.18	—							
3. HANEs^b^	5.44	2.18	0.35*	−0.11	—						
4. LANEs^b^	5.05	2.24	−0.11	0.41**	0.56***	—					
5. Affect regulation function^c^	5.05	1.38	−0.25	−0.17	0.22	0.10	—				
6. Anti‐dissociation function^c^	2.52	1.87	−0.32*	0.45**	−0.24	0.29	−0.08	—			
7. Lifetime NSSI frequency	292.95	549.11	−0.26	0.17	−0.02	0.25	0.20	0.39*	—		
8. Negative affect (PANAS‐N)	28.30	8.51	−0.46**	0.36*	0.02	0.44**	0.10	0.15	0.35*	—	
9. History of suicide attempt^d^	0.25	0.49	−0.05	0.08	0.15	0.32*	0.02	0.33*	0.28	0.22	—

*Note:* Values represent Spearman rank‐order correlations among person‐level variables. HANE, LANE, pain, and numbness scores were averaged across NSSI episodes during the EMA period.

Abbreviations: HANEs, high‐arousal negative emotions; LANEs, low‐arousal negative emotions; PANAS‐N, Negative Affect subscale of the Positive and Negative Affect Schedule.

^a^ Intensity score retrospectively rated between 0 and 10 during non‐suicidal self‐injurious behavior.

^b^ Composite intensity score retrospectively rated between 0 and 10 immediately preceding non‐suicidal self‐injurious behavior.

^c^ Function endorsement as measured by the Inventory of Statements about Self‐Injury.

^d^ Binary history where 0 = *has never attempted suicide* and 1 = *has attempted suicide at least once*.

*p < 0.05, **p < 0.01, ***p < 0.001.

### 2.2 Pain and Numbness

Two multilevel models were used to predict physical pain and numbness during NSSI episodes from arousal‐based negative emotion composites. In these models, HANE and LANE scores reflected participants' retrospective ratings of how intensely they experienced specific emotions immediately before self‐injuring, whereas pain and numbness reflected retrospective ratings of physical sensations during the same NSSI episode. Models separated episode‐level deviations from person‐level averages of HANEs and LANEs to distinguish within‐ and between‐person associations.

As shown in Table 4, the multilevel model predicting physical pain revealed both within‐ and between‐person associations. At the within‐person level, higher‐than‐usual LANEs relative to participants' own averages immediately before NSSI were significantly associated with greater physical pain during the episode. At the between‐person level, individuals who reported higher average HANEs across the study reported experiencing greater pain during NSSI, whereas individuals with higher average LANEs reported lower pain during the behavior. Momentary HANEs were not a significant predictor of physical pain during NSSI.

**TABLE 4:** Fixed effects from multilevel models predicting physical pain and numbness during NSSI episodes.

Outcome	Predictor	*b*	SE	95% CI	*p*
Pain^a^	HANEs^b^ (within‐person)	0.010	0.106	[−0.200, 0.220]	0.926
	LANEs^b^ (within‐person)	0.363	0.116	[0.159, 0.619]	0.001
	HANEs^b^ (between‐person)	0.556	0.135	[0.283, 0.829]	< 0.001
	LANEs^b^ (between‐person)	−0.383	0.131	[−0.647, −0.119]	0.006
Numbness^a^	HANEs^b^ (within‐person)	0.154	0.109	[−0.063, 0.371]	0.162
	LANEs^b^ (within‐person)	−0.031	0.119	[−0.268, 0.206]	0.739
	HANEs^b^ (between‐person)	−0.587	0.191	[−0.975, −0.200]	0.004
	LANEs^b^ (between‐person)	0.824	0.187	[0.515, 1.273]	< 0.001

*Note:* Separate linear mixed‐effects models were estimated for pain and numbness. Within‐person predictors reflect episode‐level deviations from each participant's own mean, between‐person predictors reflect participant‐level averages across NSSI episodes.

Abbreviations: HANEs, high‐arousal negative emotions; LANEs, low‐arousal negative emotions.

^a^ Intensity score retrospectively rated between 0 and 10 during non‐suicidal self‐injurious behavior.

^b^ Composite intensity score retrospectively rated between 0 and 10 immediately preceding non‐suicidal self‐injurious behavior.

For physical numbness during NSSI episodes, only between‐person emotional differences emerged as significant predictors (see Table 4). Individuals who reported higher average LANEs overall experienced greater numbness during NSSI, whereas those who reported higher average HANEs experienced significantly less numbness. Neither momentary fluctuations in HANEs nor LANEs immediately preceding NSSI were significantly associated with numbness during the episode.

## 3 Discussion

The present study examined whether high‐arousal negative emotions (HANEs; overwhelmed, anxious/afraid, angry, frustrated) and low‐arousal negative emotions (LANEs; sad, lonely, empty, hurt/rejected) reported before NSSI were differentially associated with physical pain and numbness during NSSI episodes in daily life using ecological momentary assessment (EMA) methodology. Overall, hypotheses were partially supported. As expected, average HANEs and LANEs before NSSI were positively correlated, suggesting that individuals who tended to report higher levels of one form of negative affect before NSSI also tended to report higher levels of the other. However, contrary to expectations, average physical pain and physical numbness during NSSI showed no significant association. This finding suggests that pain and numbness may reflect distinct somatosensory experiences during NSSI rather than opposite ends of a single continuum (Flowers et al. 2021; Westfall and Veilleux 2026).

Primary model findings were also partially consistent with hypotheses. At the between‐person level, findings generally aligned with predictions: individuals who reported higher average HANEs before NSSI reported greater pain and lower numbness during NSSI, whereas individuals who reported higher average LANEs before NSSI reported greater numbness and lower pain. In contrast, within‐person findings were less consistent with hypotheses. Episodes preceded by higher‐than‐usual LANEs were associated with greater, rather than lower, pain during NSSI, and neither momentary HANEs nor momentary LANEs significantly predicted numbness during NSSI. Thus, the hypothesized arousal‐based pattern was most clearly supported at the between‐person level, whereas episode‐level associations were more limited and, in the case of LANEs and pain, contrary to prediction.

At the bivariate level, both average LANE intensity before NSSI and anti‐dissociation function endorsement were positively associated with lifetime history of suicide attempt. Although these associations were cross‐sectional at the person level and cannot establish directionality or causal risk, they suggest that LANE and dissociation‐related motives may characterize a subgroup of individuals with more severe clinical histories. This interpretation is consistent with prior work linking anti‐dissociation functions of NSSI to suicide‐related outcomes. Specifically, anti‐dissociation function endorsement has been associated with a shorter transition time from NSSI onset to suicide attempt, and anti‐dissociation functions have shown among the strongest associations with lifetime suicide attempt history relative to other NSSI functions (O'Loughlin et al. 2021; Paul et al. 2015). Together, these findings suggest that experiences involving emptiness, loneliness, disconnection, and the use of NSSI to interrupt numbness or feel real may be clinically meaningful markers of risk among individuals who self‐injure.

Contrary to some prior work implicating diminished pain during NSSI in suicide risk, lower physical pain during NSSI was not associated with lifetime suicide attempt history in the present study (Ammerman et al. 2016; Franklin et al. 2012; Hepp et al. 2026). It may be that reduction in NSSI pain over time may be what confers elevated suicide risk, as it could indicate an individual's key coping skill is losing perceived efficacy. Future studies with larger samples and prospective designs should examine whether numbness‐related NSSI experiences, anti‐dissociation motives, and diminished pain sensitivity independently or interactively predict suicidal thoughts, suicide attempts, or escalation in NSSI severity.

At the between‐person level, individuals who reported higher average HANEs before NSSI episodes also reported greater physical pain during NSSI. This finding suggests that individuals who typically experience NSSI in the context of heightened arousal may experience self‐injury as more physically painful, in alignment with previous cross‐sectional research finding that people who reported generally feeling more intense emotional distress before NSSI also reported generally experiencing higher levels of pain during NSSI itself (Carpenter et al. 2023). One possibility for this finding is that high‐arousal negative emotions increase physiological activation or attentional engagement with the body, making pain more salient during NSSI (Esteve and Camacho 2008; Wiech and Tracey 2009). This interpretation aligns with affective models suggesting that high‐arousal negative states may be especially difficult to tolerate and may increase urgency to engage in behaviors that provide immediate relief (see Chapman et al. 2006). In this context, physical pain may function as a salient bodily stimulus that competes with or redirects intense emotional arousal.

In contrast, low‐arousal negative emotions appeared particularly relevant to physical numbness during NSSI. Individuals who reported higher average LANEs before NSSI episodes reported greater physical numbness during NSSI. This finding may reflect a dissociative or disconnection‐based pathway to NSSI, in which emotions such as emptiness, loneliness, sadness, and rejection are associated with reduced bodily sensation or feeling unreal, disconnected, or emotionally shut down (Dewe et al. 2016; Hagan et al. 2019; Price and Thompson 2007). Consistent with this interpretation, anti‐dissociation function endorsement was positively associated with physical numbness at the bivariate level. Together, these findings suggest that physical numbness during NSSI may be especially relevant for individuals who use self‐injury to interrupt dissociation, restore sensation, or feel physically present.

The distinction between within‐person and between‐person findings was also important. Momentary increases in LANEs relative to a participant's own pre‐NSSI average were associated with greater physical pain during the corresponding NSSI episode. However, at the between‐person level, higher average LANEs were associated with lower pain and greater numbness, suggesting that LANEs may operate differently across levels of analysis. Within a given person, episodes preceded by unusually high LANEs may be experienced as more painful. However, individuals who generally experience higher levels of these emotions before NSSI may be more likely to experience numbness or reduced pain during self‐injury. Thus, momentary low‐arousal distress may intensify pain during specific episodes, whereas chronic or person‐level low‐arousal distress may be linked to broader patterns of disconnection, dissociation, or diminished bodily sensation.

Interestingly, numbness was predicted primarily by between‐person differences rather than momentary deviations in pre‐NSSI emotion. Neither momentary HANEs nor momentary LANEs significantly predicted numbness during NSSI. This may indicate that physical numbness during NSSI is less tied to short‐term emotional fluctuations immediately before a given episode and more reflective of stable individual differences in affective experience, dissociation, or NSSI function. In other words, some individuals may be generally more prone to experiencing NSSI in the context of emotional and physical disconnection, whereas others may experience NSSI as more painful and arousing. Future work should examine whether traits such as dissociation proneness, interoceptive awareness, body awareness, or emotional numbing explain these person‐level differences.

These findings also have implications for understanding NSSI functions. Although affect regulation and anti‐dissociation are both considered intrapersonal (i.e., self‐reinforcing) functions of NSSI, the present results suggest that they may correspond to different emotional and somatic experiences (Nock and Prinstein 2004; Klonsky and Glenn 2009). The association between anti‐dissociation function endorsement and physical numbness supports the clinical relevance of distinguishing these motives, as individuals who self‐injure to reduce intense emotional arousal may require different intervention strategies than individuals who self‐injure to feel something when emotionally or physically numb.

Clinically, these findings highlight the importance of assessing bodily experiences during NSSI, not only emotional antecedents or behavioral functions. Standard NSSI assessment often focuses on why an individual self‐injures, what emotions precede the behavior, and whether the behavior reduces distress. The present findings suggest that clinicians may also benefit from asking directly about physical pain, physical numbness, dissociation, and bodily awareness during NSSI. High‐arousal pathways may be best addressed through distress tolerance, emotion regulation, and arousal‐reduction strategies, whereas low‐arousal or numbness‐related pathways may require sensory‐based alternatives to NSSI that serve to increase arousal and strategies for safely augmenting connection to the body.

Several limitations should be considered. First, the sample was relatively small and included 143 NSSI episodes from 40 participants, limiting statistical power and the ability to test more complex models, such as interactions between emotion type and NSSI function. Second, this study used secondary analyses and the sample size was determined by the original study design rather than an a priori power analysis for the present aims. Additionally, between‐person effects reflect individual‐difference associations across the EMA period and should not be interpreted as temporal or causal effects. Third, although EMA reduces some forms of recall bias, ratings of emotions before NSSI and physical sensations during NSSI were still reported retrospectively after the episode occurred. As a result, participants' reports of pre‐NSSI emotion, pain, and numbness may have been influenced by memory, current mood, or interpretation of the episode. Fourth, the EFA was used as an exploratory and descriptive tool to inform composite construction rather than as a confirmatory measurement model. Although the low‐arousal negative emotion factor was relatively coherent, high‐arousal emotions separated into avoidance‐ and approach‐related factors. Though these were combined into a single HANE composite for theoretical reasons, future research should examine whether avoidance‐related and approach‐related HANEs show distinct associations with pain and numbness during NSSI. Moreover, we were unable to account for NSSI method, injury severity, or body location, all of which may influence pain and numbness ratings. Finally, the exclusion of individuals above moderate suicide risk may limit generalizability to higher‐acuity clinical populations.

Future research should extend this work in several directions. Larger EMA studies could examine whether specific NSSI methods differ in their associations with pain and numbness, as method and corresponding severity characteristics may influence physical sensation during self‐injury. Additionally, research should test whether dissociation, interoceptive awareness, or body awareness mediates associations between LANEs and physical numbness. It may also be useful to examine whether NSSI functions moderate links between pre‐NSSI emotion and physical sensation, such that anti‐dissociation motives strengthen associations between LANE and numbness, whereas affect regulation motives strengthen associations between HANE and pain or relief. Lastly, future work with larger samples should examine whether demographic characteristics, baseline clinical severity, NSSI method, and NSSI severity account for or moderate between‐person associations.

## 4 Conclusion

Overall, the present study suggests that NSSI is not only an affect regulation behavior, but also an embodied behavior characterized by distinct physical sensations. High‐ and low‐arousal negative emotions may correspond to different somatic experiences during NSSI, with HANEs linked more closely to pain and LANEs linked more closely to numbness and anti‐dissociation processes. These findings underscore the value of integrating emotional, somatic, and functional models of NSSI. By attending to both what individuals feel emotionally before NSSI and what they feel physically during NSSI, researchers and clinicians may better identify distinct pathways through which self‐injury is initiated, maintained, and potentially treated.

## Author Contributions

**Michelle K. Hiner:** conceptualization, writing – original draft, methodology, formal analysis, visualization. **Evan M. Kleiman:** writing – review and editing, formal analysis. **Teresa M. Leyro:** writing – review and editing, formal analysis. **Amy Kranzler:** funding acquisition, investigation. **Edward A. Selby:** writing – review and editing, supervision, project administration, methodology, resources.

## Funding

This research was supported by the National Science Foundation (Grant No. 1211079) and the American Psychological Association (dissertation grant awarded to A. Kranzler).

## Ethics Statement

This study used de‐identified data from a previously approved study. The original study procedures were approved by the Rutgers University Institutional Review Board (Protocol #: 14‐185Rc).

## Consent

All adult participants provided informed consent, and participants under the age of 18 provided informed assent with parent/guardian consent.

## Conflicts of Interest

The authors declare no conflicts of interest.

## Data Availability

The data that support the findings of this study are available from the corresponding author upon reasonable request.
